# Analysis of Behaviors of the Railway Subgrade with a New Waterproof Seal Layer

**DOI:** 10.3390/ma15031180

**Published:** 2022-02-03

**Authors:** Wubin Wang, Zhixing Deng, Yunbin Niu, Yandong Li, Zhichao Huang, Minqi Dong, Qian Su

**Affiliations:** 1 National Engineering Research Center of Geological Disaster Prevention Technology in Land Transportation, Southwest Jiaotong University, Chengdu 611731, China; wangwubin1896@163.com; 2 School of Civil Engineering, Southwest Jiaotong University, Chengdu 610031, China; 2020210063@my.swjtu.edu.cn (Z.D.); yunbinniu@my.swjtu.edu.cn (Y.N.); swjtu1896dd@163.com (Y.L.); huangzhichao1896@163.com (Z.H.); swjtu1896d00@163.com (M.D.)

**Keywords:** railway subgrade bed, polyurethane mixture, waterproof performance, model test, field test

## Abstract

This study proposes a new waterproof sealing layer to reduce the impact of water on subgrade beds. The proposed waterproof sealing layer is composed of a polyurethane adhesive (PA) mixture, which aims to control interlaminar deformation and prevent seepage. A variety of laboratory tests were first performed to analyze the attenuation characteristics and mechanical properties of various polyurethane polymer (PP)-improved gravel mixtures under thermohydraulic coupling effects. In addition, a waterproof performance model test of the PP-improved gravel layer was conducted to investigate its waterproof and drainage performance and hydraulic damage mechanism. Finally, the feasibility and effectiveness of the surface structure of the waterproof drainage subgrade bed containing the PA mixture was tested in combination with the treatment project of the Ciyaowan station of the Baoshen heavy-haul railway. According to the experimental and model results, (1) the waterproof layer containing the polyurethane mixture exhibited satisfactory stiffness, elasticity and flexibility. The waterproof layer containing the polyurethane mixture also controlled the deformation between layers, and its mechanical properties remained stable. (2) The waterproof layer with the dense polyurethane mixture performed well in terms of the waterproof aspect, and no infiltration occurred under cyclic load (3). Long-term field monitoring revealed that the effect of the implementation of a PP-improved gravel layer to treat mud pumping was remarkable. The settlement of the PP-improved gravel layer only reached 13.21 mm, and the settlement remained stable in the later stage.

## 1. Introduction

In recent years, the waterproof layer has been found that to suffer many defects, e.g., cracks, breakage, pulverization and other diseases, when applied in the ballastless track of high-speed railway subgrade surfaces [1,2,3]. During the operation process, different degrees of hydraulic damage to the subgrade surface layer have been reported [4,5,6], especially in high-rainfall areas. These distresses mainly result from insufficient consideration of the specific operating environment and performance of the ballastless track subgrade [7,8,9]. The subgrade fine particle composition will cause subgrade bed deformation, mud boiling and other diseases when encountering water, which will affect the normal and safe operation of high-speed railways and increase the maintenance costs for the national transportation department. Therefore, ensuring the stability of the subgrade has important economic and environmental benefits.

Many scholars have performed studies on the hydraulic damage to the subgrade bed surface. In these studies, the method to improve water stability and prevent erosion has attracted considerable attention [10,11,12,13]. In the present work, the waterproof sealing layer has been considered as an effective way to control interlayer deformation and prevent seepage to reduce hydraulic damage [14]. Thus, dense asphalt concrete has been employed as the waterproof sealing layer [15,16]. It has been verified in the field that the asphalt concrete layer attains acceptable waterproof performance to improve the mechanical response of the subgrade bed surface [17,18,19]. However, asphalt concrete still exhibits certain shortcomings, such as severe segregation and cracking [19,20,21].

Alternatively, PA, as a new type of cementation material, exhibits the potential to be employed as the waterproof sealing layer in the subgrade bed surface [22,23,24,25]. In this study, we proposed a PP-improved gravel mixture as an innovative waterproof sealing layer. To meet the multifunctional composite structure requirements of the subgrade bed surface in a high-rainfall area, a series of laboratory tests considering the thermohydraulic coupling effects was performed. Next, waterproof model tests were conducted to verify the effectiveness of the PP-improved gravel layer.

Finally, combined with the practical engineering case of the Baoshen heavy-haul railway, the construction feasibility of the PP-improved gravel mixture as the waterproof sealing layer of the subgrade bed surface and the effectiveness of the PP-improved gravel mixture in subgrade bed disease treatment were verified.

## 2. Materials and Methods

### 2.1. Materials

#### 2.1.1. Graded Crushed Stone

**Figure 1:**
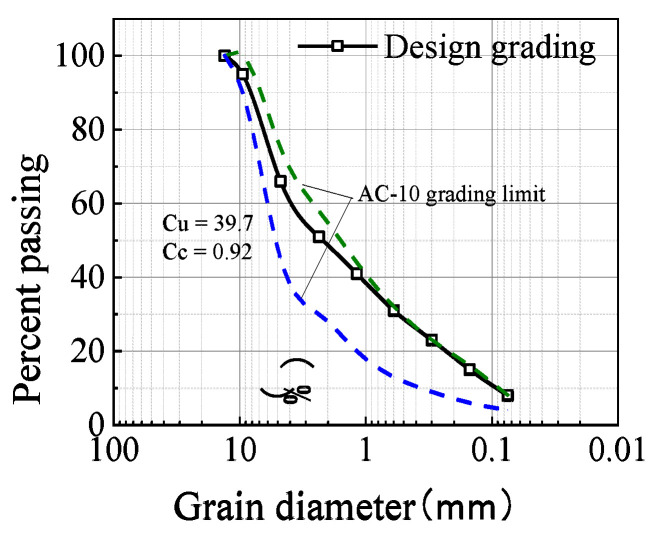
Design grading curve.

The gradation structure of stable crushed stone is generally divided into three types: (1) suspension dense structure, (2) skeleton dense structure and (3) skeleton pore structure. In [26,27,28], it is shown that the PP-improved gravel mixture was applied with a suspension dense structure, which required the thickness of the waterproof sealing layer be maintained between 3 and 5 cm for a low porosity state and the maximum aggregate diameter not exceed 1/2~1/3 of the thickness for a compacted requirement [25]. Research shows that AC-10 mineral grading can form a suspended dense structure, which has good water resistance performance and anti-cracking properties and is suitable as a waterproof anti-cracking functional layer. Finally, referring to the grading control range of continuous dense fine-grained asphalt mixture AC-10, the design grading curve of the sample is determined, as shown in Figure 1.

#### 2.1.2. PA Mixtures

In this research, the PA mixtures are divided into group A, which is a polyurethane prepolymer produced by the polymerization of isocyanate and oligomer polyol, and group B, which is a mixture of a curing agent, active agent and thickening agent. Both groups should be kept sealed because they will deteriorate when they are exposed to air for an extended period of time [25]. When using the mixtures, according to an A: B mass ratio of 4:1, at room temperature (less than 60 °C), they must be fully mixed and used as soon as possible. In addition, the PA mixture must offer high corrosion, cold and UV resistance levels. The relevant parameters of the polyurethane raw materials are listed in Table 1. The performance indexes of polyurethane adhesive used in the test in this paper meet the relevant parameter indexes of polyurethane adhesive in Table 1 so as to make the PP-improved gravel layer meet the corresponding functional characteristics.

#### 2.1.3. The Mechanical Property of PP-Improved Gravel Mixture

**Figure 2:**
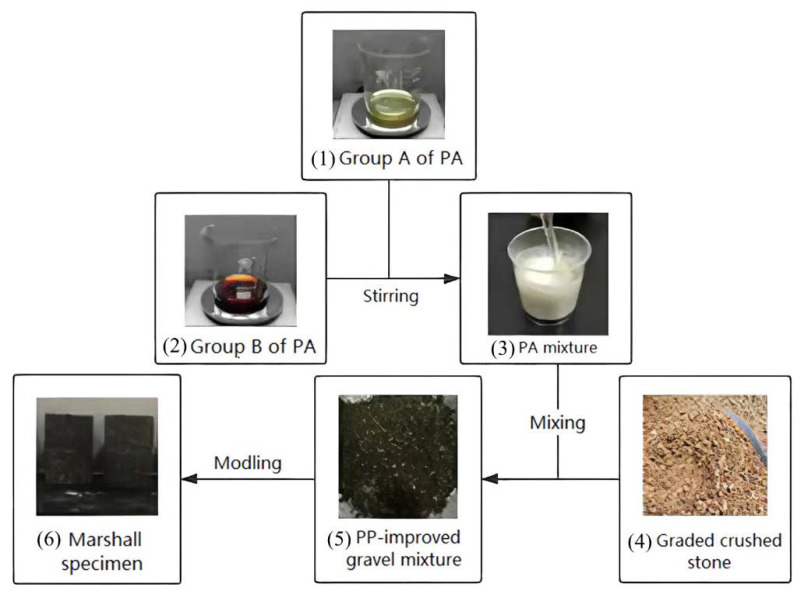
Process flow of preparation of the PA mixtures PP-improved gravel mixture.

Figure 2 shows the polyurethane gelling agent samples of groups A and B and how they become PA, PP-improved gravel mixture and Marshall specimen. In addition, the PA mixture should initially maintain the flowing state for a certain period before construction.

### 2.2. Laboratory Tests

**Figure 3:**
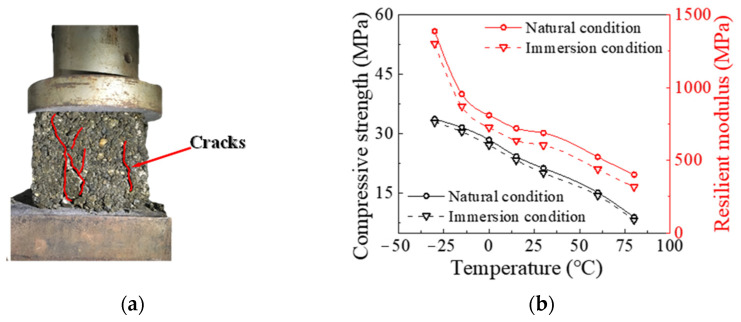
Laboratory test phenomena and results: (**a**) crack in compression test of the PP-improved gravel specimens, and (**b**) variation in the compressive strength and resilient modulus of the PP-improved gravel specimens.

In this section, a series of tests involving two groups of experiments with temperatures ranging from −30 °C to 80 °C were conducted to determine the water-induced damage and attenuation characteristics of the PP-improved gravel layer under the coupling effects of the load, water seepage and temperature. In one group, the PP-improved gravel mixtures were tested under natural conditions and buried in crushed stone outdoors for 2 months. In the other group, the PP-improved gravel mixtures were tested in underwater immersion conditions, whereby they were continuously soaked in water for 48 h. In this test, the PA mixture content remained constant at 8% [25]. The test method used in the experiments was based on the design practices of highway engineering [29]. Cube-shaped PP-improved gravel specimens with a side length of 150 mm were placed in the temperature control box of a compressive strength test system for 30 min at a given temperature, and the applied load was then gradually increased at a loading rate of 0.5 kN/s. Figure 3a shows the failure mode of the specimens and the test results are shown in Figure 3b.

Figure 3b shows the trend of the compressive strength and resilient modulus of the PP-improved gravel specimens versus the temperature. The compressive strength decreased from 33.7 to 8.9 MPa with increasing temperature under natural conditions. In addition, the resilient modulus decreased from 1387.0 to 400.7 MPa, a decrease of 71.1%. In underwater immersion conditions, the compressive strength of the PP-improved gravel specimens decreased by 0.7 to 1.4 MPa, and the resilient modulus decreased by 85 MPa. This indicates that the PP-improved gravel specimens are sensitive to external temperature changes. However, the mechanical properties of the PP-improved gravel specimens remain stable. The PP-improved gravel specimens still exhibit outstanding water resistance under the considered thermohydraulic coupling effects.

To investigate the waterproof performance and deformation characteristics of the PP-improved gravel layer, a model box made of steel that controls the water inflow and outflow was manufactured in a size of 58 cm in length, 52 cm in width and 65 cm in height respectively. The thickness of the bottom steel plate is 2.5 cm, while the thickness of the remaining steel plates equals 1.5 cm. Both sides of the model box are fixed on a column with clamps to prevent shaking during the loading process. After assembling the servo board and partition at the opening position, a valve is connected to form a drainage pipe. To reduce the influence of the model box on the force state of the structure boundary, two extruded plates with a thickness of 1 cm are pasted on the left and right sides of the model box. The influence of the side boundary on the structural deformation is monitored by placing a soil pressure cell on the side. The bottom of the model box is regarded as a rigid boundary. Therefore, it is not necessary to place extruded plates on the bottom of the model box. Additionally, soil pressure sensors, pore pressure sensors and acceleration sensors were embedded at the bottom, middle and top of the PP-improved gravel layer, respectively, for monitoring the stress and deformation of each layer. Settlement plates were placed at the bottom and top of the PP-improved gravel layer. Finally, strain gauges were affixed to the center and lateral sides of the loading plate and displacement and acceleration sensors were placed on the surface of the settlement plate.

**Figure 4:**
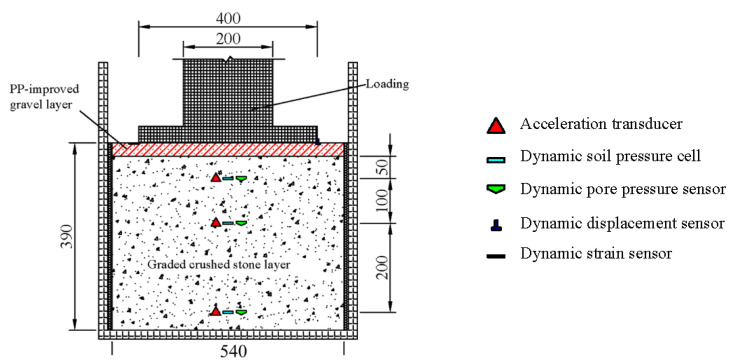
Model design.

In this study, three experimental models, including the graded aggregates (GA) model, dense polyurethane graded aggregates (PU-GA) model, and permeable polyurethane graded aggregates (PPU-GA) model, were designed. In all three models, the subgrade bed was divided into a 360-mm graded crushed stone layer, a plastic cloth, and a 1-cm gravel cushion from bottom to top, with the distinction that there is a 30-mm PP-improved gravel stone layer in PU-GA model, a 30-mm permeable PP-improved gravel stone layer in the PPU-GA model and no PP-improved gravel stone layer in the GA model. The model box and the sensor distribution are shown Figure 4.

**Figure 5:**
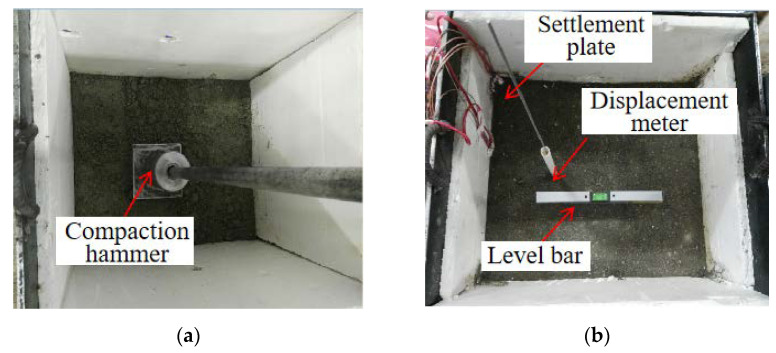

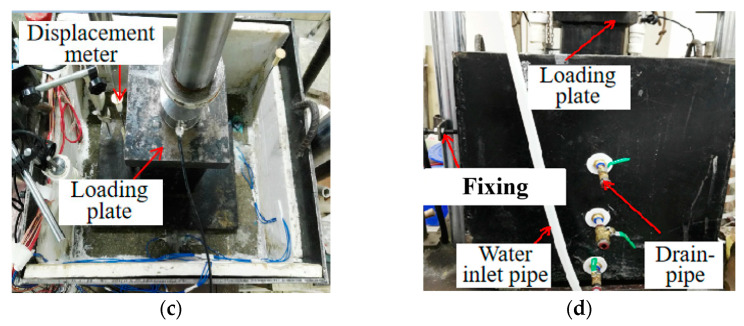
Process flow of laboratory test model design: (**a**) filling Gravel layer, (**b**) Filling waterproof layer, (**c**) placing loading/monitoring devices and (**d**) complete model.

Compared to the PU-GA model, the GA model contained no PP-improved gravel layer. Rainfall was simulated by pouring water on the surface of all models. If all the water had infiltrated the sub-base during cyclic dynamic loading, additional water was continuously poured on the surface of the models until the model failed. After the PP-improved gravel layer was added in the PU-GA model, a layer consisting of dense polyurethane graded gravel was placed directly on the surface of the model box to prevent water from penetrating the graded gravel layer along the sidewalls. The filling process of all materials was completed strictly according to the compactness requirements. The filling process is shown in Figure 5.

The test load amplitude is converted according to the stress equivalence principle [30]. Numerical calculations and field tests have suggested that the dynamic stress of the ballastless track subgrade surface should range from 10 to 20 kPa [10]. Liu Gang et al. [31] analyzed the dynamic stress distribution of the subgrade surface of a ballastless track combined with a field test and recommended 40 kPa as the design value of the dynamic stress amplitude of the subgrade surface. According to the stress equivalence principle, the static load amplitude is calculated as 5 kN. The test loading process is divided into two stages. In the first stage, the static load was increased from 0 to 5 kN at 1-kN intervals. In the second stage, a dynamic load was applied. In this paper, the loading range was from 0.5 to 4.5 kN, and the loading frequency ranged from 3 to 5 Hz. In this study, all models were loaded 24,000 times under natural and immersion conditions.

### 2.3. Field Tests

The Ciyaowan station of the Baoshen heavy-haul railway is located in the town of Daliuta, Shenmu County, Shanxi Province. Because coal gangue filled in the bottom of the subgrade bed and coal cinder was dispersed by freight trains along the line, the subgrade bed easily became harder, while the water on the surface has a higher possibility of seeping into the subgrade, which causes the water to easily accumulate at the center of the subgrade and results in mud pumping.

However, the accuracy of the aggregate parameters provided by the quarry administration office did not meet the laboratory test requirements. The quarry aggregate gradation range is large, and the quality parameters make it difficult to accurately ensure the polyurethane crushed stone gradation requirements are met, so the average gradation range of crushed stone for site construction is revealed through indoor sieving tests, and physical and mechanical property tests of polyurethane cool gelling graded crushed stone are carried out to determine the optimal glue admixture, which in turn provides theoretical guidance for site construction. Therefore, in this study, several tests were conducted to determine the waterproof performance of the PP-improved gravel layer.

The waterproof performance of the PP-improved gravel layer is evaluated by the permeability coefficient and the specimen porosity. Different PP-improved gravel mixtures with the PA content ranging from 5% to 10% at 1% intervals were prepared according to the Marshall testing method, in line with the Chinese code of the permeability coefficient of asphalt concrete [32].

**Figure 6:**
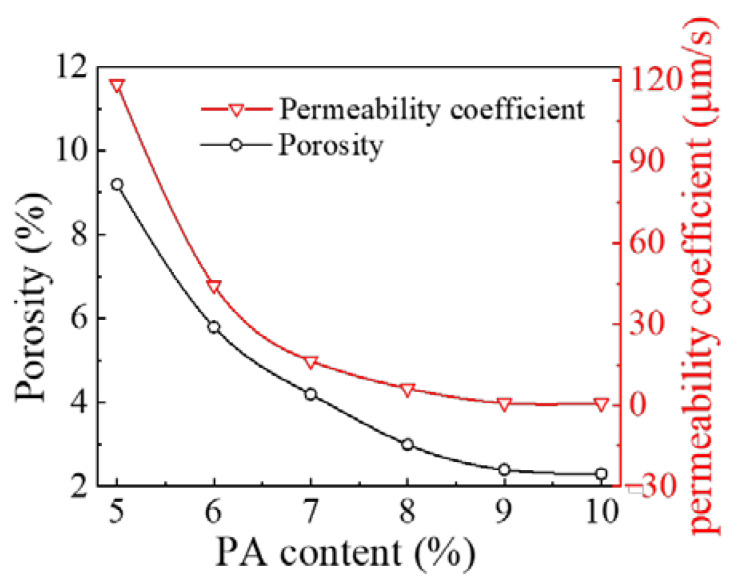
Variation in porosity and permeability coefficient with the PA mixture content.

Figure 6 shows both the water seepage test results and the measured specimen porosities. The specimens were maintained for 48h at room temperature under natural conditions, and the porosity and permeability coefficients were tested at room temperature, which illustrates the variation in the permeability coefficient of the PP-improved gravel specimens with the PA content. The permeability coefficient of the PP-improved gravel specimens tends to decrease with increasing PA content. When the PA content exceeds 8%, the permeability coefficient is slightly reduced. The permeability coefficient of the PP-improved gravel specimens is 8.3 × 10^−7^ m/s at a PA content of 9%, which reaches the impermeable level and meets the requirements of a waterproof structural layer. Furthermore, there is a similar tendency in the variation of porosity, which is slightly smoother from 9.2% to 2.3% with PA content increasing from 5% to 10%.

In conclusion, when the gravel aggregate from the nearby quarry is chosen as the raw material, a PA content of 9% satisfies the requirements of the waterproof sealing layer based on economic considerations.

**Figure 7:**
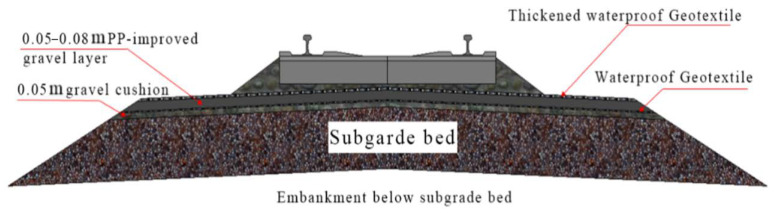
Structural drawing of the waterproof PP-improved gravel layer.

**Figure 8:**
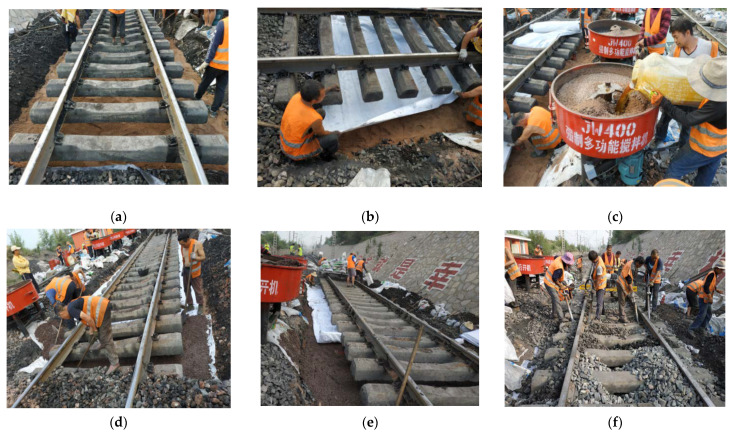
Structural drawing of the waterproof PP-improved gravel layer: (**a**) laying sand cushion, (**b**) laying bottom geotextile, (**c**) Mixing of PA, (**d**) paving PU-GA layer, (**e**) laying upper geotextile and (**f**) backfilling and tamping ballast.

The mud pumping treatment applied at this station included the addition of a new waterproof PP-improved gravel layer below the subgrade bed. Figure 7 shows the specific structure of the PP-improved gravel layer, and Figure 8 shows the whole process for improving serviceability by PU-GA, including: Figure 8a laying sand cushion, Figure 8b laying bottom geotextile, Figure 8c mixing of PA, Figure 8d paving PU-GA layer, Figure 8e laying upper geotextile and Figure 8f backfilling and tamping ballast.

**Figure 9:**
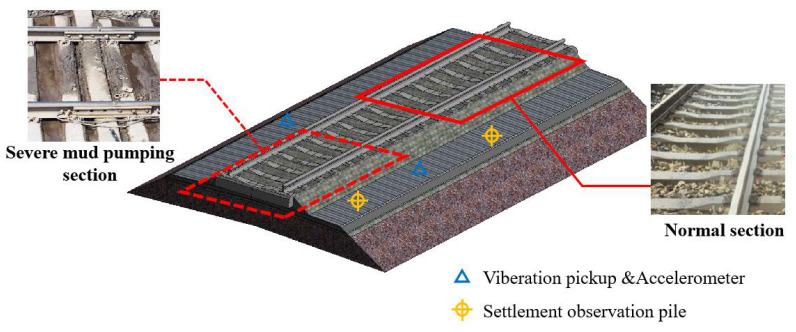
Acceleration sensor field layout.

To investigate the impact of the treatment, a model c8002 Shenhua self-provided freight train was selected for the tests, and the freight train has an axle load of 20 t, while the load is 80 t. Accelerometers were installed at the ends of sleepers along the serious mud pumping section and normal downline section to assess the vibration response of the sleepers during operation [33,34]. In addition, the treatment effect on the mud pumping phenomenon occurring in the slab track subgrade was determined, which provides a reference for the new materials applied in the mud pumping treatment, as shown in Figure 9.

## 3. Experimental Results

### 3.1. Laboratory Tests

**Figure 10:**
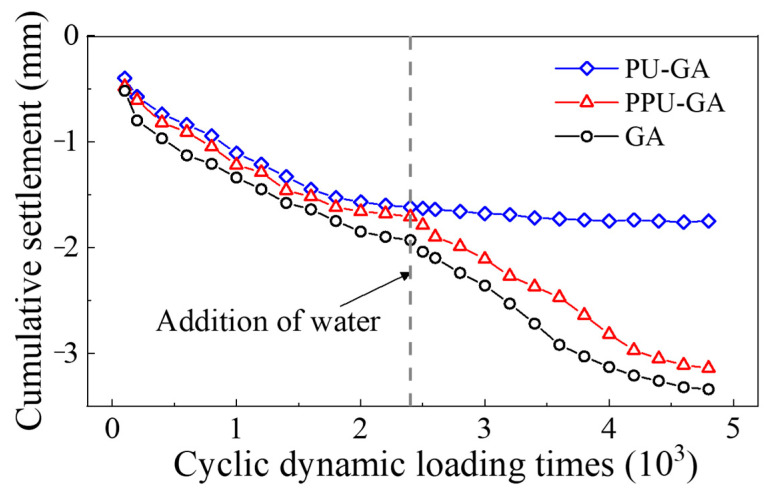
Variation in the cumulative settlement with the dynamic loading cycles.

Figure 10 shows the cumulative settlement at the surface of all the models, PU-GA, PU-GGA and GA, versus the dynamic loading cycles, which were measured by a displacement sensor on the surface of the PP-improved gravel layer. In the first stage of loading, the cumulative settlement at the surface of the PU-GA model quickly increased due to the decreasing porosity resulting from loading. With increasing dynamic loading cycles, the cumulative settlement of all models increased from 0 to 20,000 and had similar tendencies insofar as the growth rate of the cumulative settlement of those models gradually decreased and tended to stabilize after 20,000 cycles. After water was poured on the surface of the models, no water flowed out of the drain outlet on the lateral side of the PU-GA model box, and the PU-GA model structure notably prevented water from penetrating the graded crushed stone layer under cyclic dynamic loading application. However, the PPU-GA and GA models failed to prevent water from penetrating under cyclic dynamic loading application. Moreover, the stiffness of the graded crushed stone used as the filler material of the lower subgrade bed layer decreased and eventually failed. The cumulative settlement of the PPU-GA and GA models increased with increasing dynamic loading cycles. Therefore, the PU-GA mode should be employed to prevent water from infiltrating the graded crushed stone layer.

**Figure 11:**
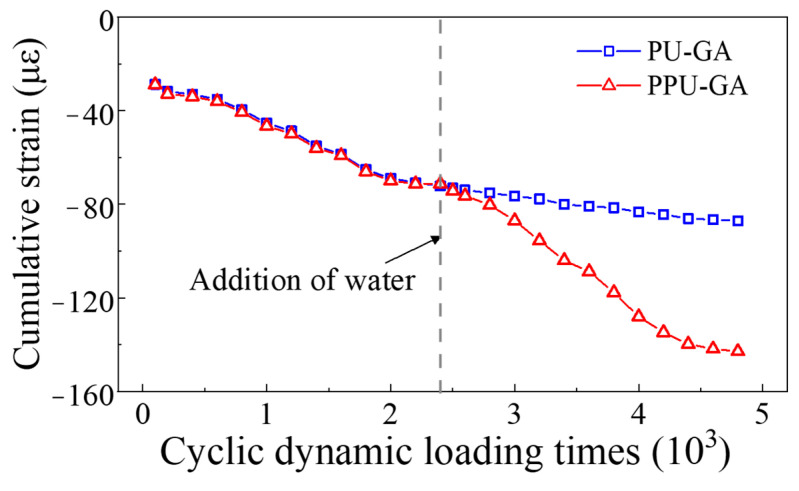
Variation in the cumulative strain with the dynamic loading cycles.

As Figure 11 shows, the variation in the cumulative strain of the PP-improved gravel layer, which was measured by a strain gauge on the surface of the PP-improved gravel layer, is similar to that in the cumulative settlement. Initially, the cumulative strain of the PU-GA model slightly increased with the dynamic loading cycles. With increasing dynamic loading cycles, the growth rate of the cumulative strain of the PU-GA model quickly increased. After water was poured on the surface, the cumulative strain of the PU-GA model gradually increased and finally tended to remain constant. According to the test results, no water infiltrated the graded crushed stone layer, which demonstrated that the PU-GA model was effective in decreasing the cumulative strain. However, in regard to the PPU-GA model, the cumulative strain of the graded crushed stone layer continuously increased. After water was poured on the surface, the cumulative strain of the PPU-GA model sharply increased. When loaded 40,000 times, the cumulative strain of the PPU-GA model was almost twice that of the PU-GA model.

**Figure 12:**
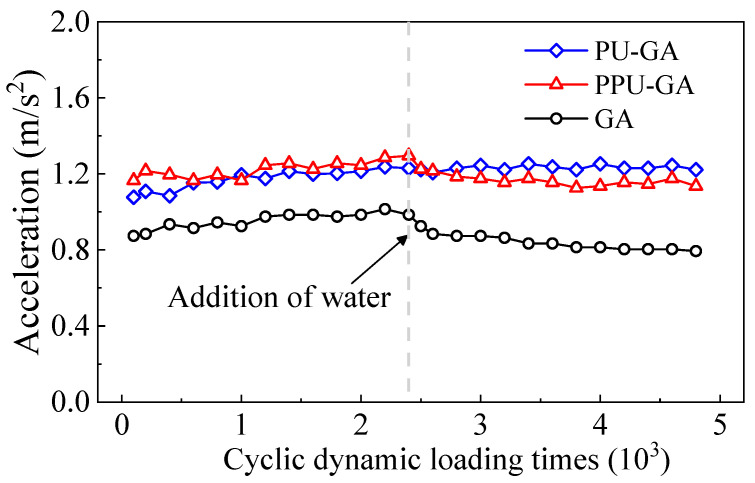
Variation in the acceleration with the dynamic loading cycles.

Figure 12 shows the variation in the acceleration of the upper graded crushed stone layer with the dynamic loading cycles. Before water was poured on the surface of all models, the acceleration of all models tended to remain stable after 10,000 loading cycles. The slight fluctuations in acceleration during this period were caused by the change in contact between the PP-improved gravel layer and graded crushed stone layer. The acceleration amplitude of the GA model was smaller to that of the PU-GA and PPA-GA models. When water was poured on the surface of all models, water penetrated and softened the grade crushed stone layers in the PPU-GA and GA models. The stiffness and acceleration amplitude of the graded crushed stone layer was marginally reduced. However, the acceleration amplitude of the PU-GA model remained constant with increasing dynamic loading cycles.

**Figure 13:**
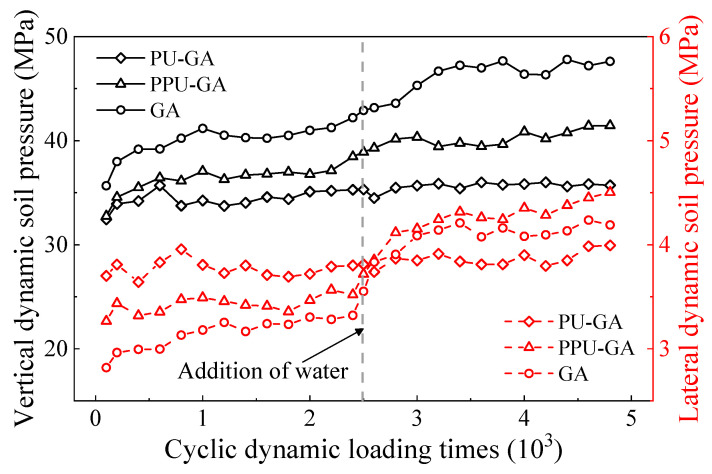
Variation in vertical and lateral dynamic soil pressure with the dynamic loading cycles.

Figure 13 shows the vertical and lateral dynamic soil pressures of all models with the dynamic loading cycles. As shown in the left axis, the vertical and lateral dynamic soil pressures of all models slightly increased at first and tended to remain stable after 10,000 loading cycles. In addition, the vertical and lateral dynamic soil pressures of the GA model were higher than those of the PU-GA and PPU-GA models before water was poured on the surface of all models. After water was poured on the surface of all models, the vertical dynamic soil pressure of the GA model remained constant. However, the vertical dynamic soil pressure of the PU-GA and PPU-GA models increased. The right axis in Figure 13 shows the variation in the lateral dynamic soil pressure with the dynamic loading cycles. The lateral dynamic soil pressure increased with increasing dynamic loading cycles from 0 to 50,000, especially after water was poured on the surface. Similar to the trend of the vertical dynamic soil pressure, the lateral dynamic soil pressure of the PPU-GA and GA models notably increased after water was poured on the surface and then slightly increased until a steady state was reached.

**Figure 14:**
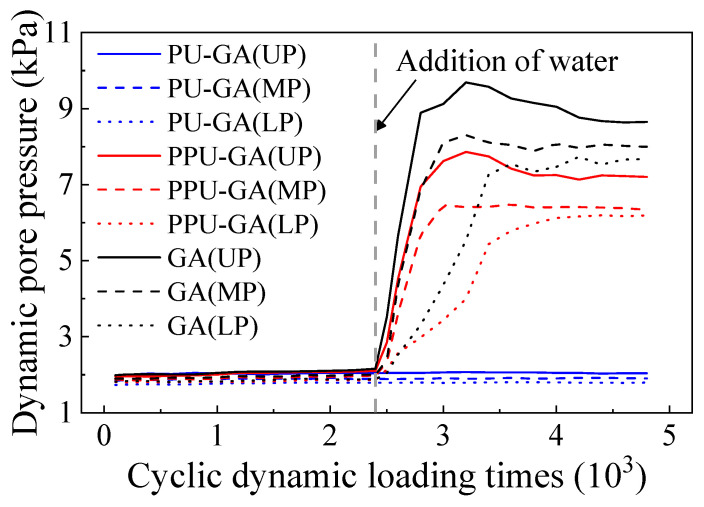
Variation in the dynamic pore pressure with the dynamic loading cycles.

The trend of the dynamic pore pressure at the upper, middle and lower positions of all models with the dynamic loading cycles is shown in Figure 14. The dynamic pore pressure of the PU-GA model remained stable before and after water was poured on the surface of the model. The dynamic pore pressure at the upper position of the PU-GA model reached approximately 2 kPa, and the dynamic pore pressure decreased with the depth. When the upper graded crushed stone layer was emplaced, sufficient fine particles were filled in the graded crushed stone layer to ensure that its surface was smooth. This resulted in a relatively low permeability coefficient of the graded crushed stone layer, and the dynamic pore pressure hardly dissipated. Before water was poured on the surface, the dynamic pore pressure of the PPU-GA and GA models remained constant at 2 kPa. After water was poured on the surface, the dynamic pore pressure at the upper and middle positions of the graded crushed stone layer sharply increased and gradually stabilized with increasing dynamic loading cycles. However, the dynamic pore pressure at the lower position of the model more slowly increased than at the other two positions. The primary reason for these phenomena is that the upper and middle positions of the graded crushed stone layer gradually reach the saturation state with increasing dynamic loading cycles, while the lower position of the graded crushed stone layer reaches the saturation state through seepage. In addition, obvious dissipation occurred after the dynamic pore pressure at the upper position of the model reached its maximum value, which indicates that the pore water was drained by the drainage pipe in this process.

**Figure 15:**
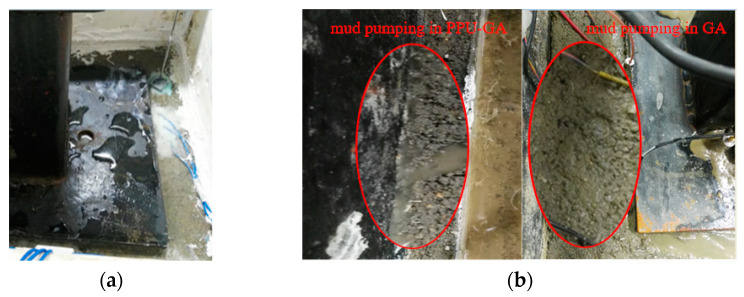
Hydraulic damage in the GA, PU-GA and PPU-GA models: (**a**) no mud pumping in the PU-GA model and (**b**) mud pumping in the PPU-GA model and GA model.

Figure 15 shows the hydraulic damage phenomena in the PU-GA, PPU-GA and GA models under dynamic loading after pouring water on the surface of the models. During the loading process, no water penetrated the graded crushed stone layer of the PU-GA model, which may prevent the occurrence of mud pumping in the ballasted track bed. This result demonstrates the excellent waterproof performance of the PU-GA model. The initial bonding strength of the PPU-GA structure is limited by the PA content in the PP-improved gravel layer during filling. Under dynamic loading application, the initial bonding strength of the interlayer gradually decreases, and the upper and lower structural layers are partially separated. After water is poured on the surface of the models, the water content in the graded crushed stone layer increases, and free water gradually accumulates between layers. With the change in dynamic pore pressure, mud pumping occurs between the graded crushed stone layer and the PP-improved gravel layer under dynamic loading. After water was poured on the surface of the model, severe mud pumping occurred in the GA model under dynamic loading without the upper waterproof sealing layer. Mud was extruded from the graded gravel layer at the opening of the loading plate due to the dynamic pore pressure increase.

### 3.2. Field Test Results

**Figure 16:**
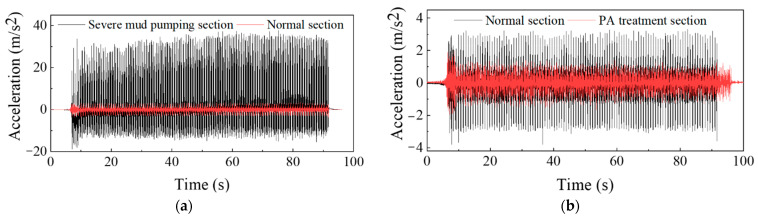
Field test results: (**a**) acceleration time history curve before PA Treatment compared with normal section when heavy-haul freight train passing through the severe mud pumping section at 80 km/h; and (**b**) acceleration time history curve after PA Treatment compared with normal section when train passing through the severe mud pumping section at 80 km/h.

Before the treatment, the acceleration time history curves of the sleepers in the serious mud pumping section and normal section are shown in Figure 16a.

As shown in Figure 16a, when the freight train passed through the severe mud pumping section, the vibration acceleration amplitude of the sleeper ranged from 20.84~33.24 m/s^2^, and the average amplitude was 30.25 m/s^2^. The vibration acceleration amplitude of the sleeper when the freight train passed through the normal section ranged from 2.67~3.25 m/s^2^, and the average amplitude was 2.53 m/s^2^. When a fully loaded freight train travels at 80 km/h, the average acceleration amplitude of the severe mud pumping section is 11.96 times that of the normal section. After the ballast excavation at the site, problems such as ballast compaction and sleeper emptying were observed in the severe mud pumping section. These problems resulted in the severe vibration of the sleepers during operation. The acceleration amplitude greatly fluctuated due to the increased sleeper displacement.

The sleeper acceleration time history curve compared with the normal section when the train is passing through the severe mud pumping section at 80 km/h is shown in Figure 16b.

**Figure 17:**
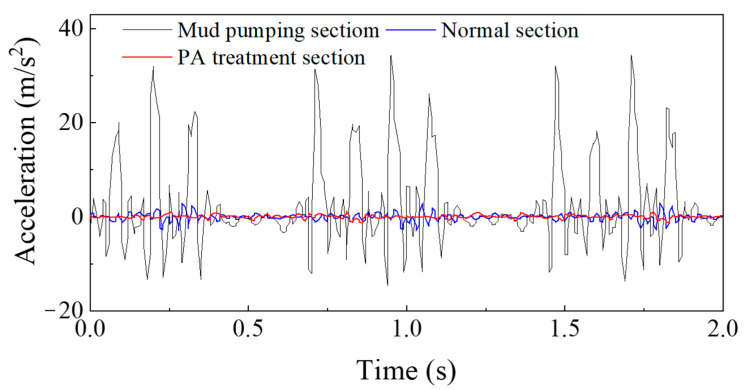
Typical acceleration time history curve in typical periods in all the working condition.

As shown in Figure 16b, the sleeper acceleration amplitude ranges from 1.22~1.28 m/s^2^, and the average acceleration is 1.25 m/s^2^, which is 0.54 times lower than in the normal section. The sleeper vibration tends to remain constant, which is similar to the normal section, with a major improvement over the sleeper vibration before the treatment. Compared to the section without the PP-improved gravel layer, the average acceleration amplitude is reduced by 2.02 times, which demonstrates the vibration reduction effect of the PP-improved gravel layer. Selecting the typical periods from 3 working conditions, the fine regulation effect can be indicated as shown in Figure 17.

In conclusion, the proposed PP-improved gravel layer with a satisfactory strength and elasticity could greatly reduce the slab track vibration. The smoothness and service life of the slab track were improved by the addition of the PP-improved gravel layer to the subgrade bed. The dynamic detection method was applied to evaluate the effect of the PP-improved gravel layer in the mud pumping treatment. The effectiveness and feasibility of the PP-improved gravel layer in the mud pumping treatment of the Baoshen heavy-haul railway were confirmed through data analysis of the dynamic detection results.

**Figure 18:**
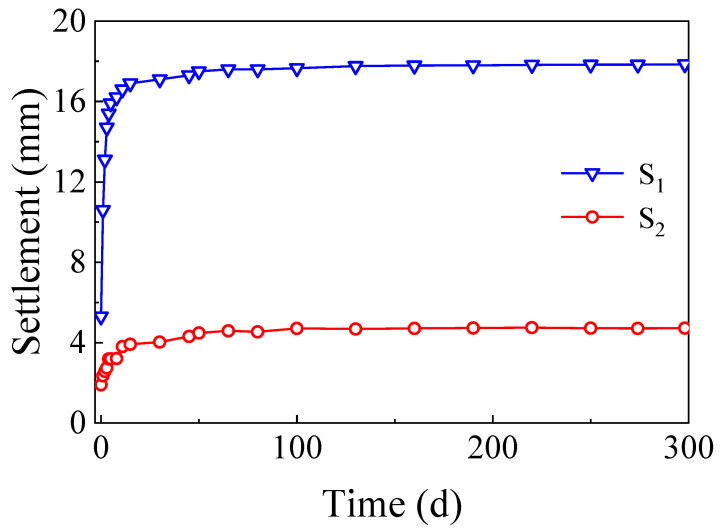
Subgrade settlement monitoring of the test section.

The field test also showed the long-term displacement in both sections to be about a year. During the construction process, two high-precision levelling sensors embedded at specific observation locations in the PP-improved gravel layer monitored the cumulative settlement. One observation location was at the bottom of the PP-improved gravel layer, which was denoted as S1, and the other observation location at the top of the PP-improved gravel layer was denoted as S2. The test results are shown in Figure 18.

As shown in Figure 18, the cumulative settlement of the subgrade bed is 17.89 mm, including a compression deformation of 13.21 mm after the addition of the PP-improved gravel layer. Settlement of the PP-improved gravel layer largely occurred within 7 days after construction and gradually stabilized over time. In addition, according to the feedback provided by on-site engineers, no mud pumping occurred after several months during the rainy season. This indicates that the addition of the waterproof PP-improved gravel layer effectively prevents mud pumping from occurring.

## 4. Conclusions

This study focused on the subgrade bed surface structure containing a new waterproof PP-improved gravel layer, The mechanical properties, waterproof properties and construction feasibility were studied by means of laboratory tests and field tests. The test results provide a meaningful reference for the treatment of surface hydraulic damage and problems of high-speed railway subgrade beds in high-rainfall areas. The following conclusions were drawn from the results of this study:

1. At a PA content of 8%, the mechanical properties of the PP-improved gravel layer satisfy the mechanical requirements of the waterproof sealing layer under the most unfavorable water immersion conditions at room temperature with a low cost;
2. The PU-GA model effectively prevents water from infiltrating the graded crushed stone layer after water immersion. However, the GA and PPU-GA models fail to prevent water penetration, and mud pumping still occurs in these models. Therefore, the waterproof sealing layer of the subgrade bed should consist of a dense PP-improved graded gravel layer;
3. The new subgrade bed surface structure containing the PP-improved gravel layer performs well in the mud pumping treatment of the Baoshen heavy-haul railway. The treatment outcomes demonstrate that the PP-improved gravel layer can be employed as a waterproof sealing layer on the subgrade surface to avoid the mud pumping phenomenon;
4. The future work of this study involves its extension into the mesoscopic field. The three- and two-dimensional distribution characteristics of the pores in polyurethane macadam specimens will be analyzed by the double peak method combined with computed tomography (CT) imaging. A 3D reconstruction algorithm of PP-improved gravel mixtures based on the obtained X-ray CT images will be proposed to establish three-dimensional numerical samples. Finally, the discrete element method (DEM) will be adopted to simulate the creep strain of PP-improved gravel mixtures.

## Figures and Tables

**Table 1:** Main performance parameters of the PA mixtures.

Parameters	Value	Parameters	Value
Density	1.2 g·cm^−3^	Surface drying time	≥0.5 h
Viscosity	3000 cP	Actual drying time	≤12 h
Elongation at failure	>2%	Tensile strength	≥12 MPa (24 h) & ≥15 MPa (7 d)
Shore hardness	40~65	Service temperature	−40 °C~90 °C

## Data Availability

Not applicable.
